# Developing a Provincial Surveillance and Support System for Childhood Cancer Survivors: Multiphase User-Centered Design Study

**DOI:** 10.2196/37606

**Published:** 2022-09-13

**Authors:** Jennifer Shuldiner, Nida Shah, Catherine Reis, Ian Chalmers, Noah Ivers, Paul Nathan

**Affiliations:** 1 Institute for Health System Solutions and Virtual Care Women's College Hospital Toronto, ON Canada; 2 Child Health Evaluative Sciences The Hospital for Sick Children Research Institute Toronto, ON Canada; 3 Pivot Design Group Toronto, ON Canada; 4 Institute of Health Policy, Management and Evaluation University of Toronto Toronto, ON Canada; 5 Department of Family and Community Medicine University of Toronto Toronto, ON Canada; 6 Division of Hematology/Oncology The Hospital for Sick Children Toronto, ON Canada

**Keywords:** design, cancer screening, childhood cancer survivor, late effects, surveillance, cancer, cancer survivor, morbidity, mortality, cancer treatment, mammogram, echocardiogram

## Abstract

**Background:**

Survivors of childhood cancer are at lifelong risk of morbidity (such as new cancers or heart failure) and premature mortality due to their cancer treatment. These are termed late effects. Therefore, they require lifelong, risk-tailored surveillance. However, most adult survivors of childhood cancer do not complete recommended surveillance tests such as mammograms or echocardiograms.

**Objective:**

In partnership with survivors, family physicians, and health system partners, we are designing a provincial support system for high-priority tests informed by principles of implementation science, behavioral science, and design thinking.

**Methods:**

Our multiphase process was structured as follows. Step 1 consisted of a qualitative study to explore intervention components essential to accessing surveillance tests. Step 2 comprised a workshop with childhood cancer survivors, family physicians, and health system stakeholders that used the Step 1 findings and “personas” (a series of fictional but data-informed characters) to develop and tailor the intervention for different survivor groups. Step 3 consisted of intervention prototype development, and Step 4 involved iterative user testing.

**Results:**

The qualitative study of 30 survivors and 7 family physicians found a high desire for information on surveillance for late effects. Respondents indicated that the intervention should help patients book appointments when they are due in addition to providing personalized information. Insights from the workshop included the importance of partnering with both family physicians and survivorship clinics and providing emotional support for survivors who may experience distress upon learning of their risk for late effects. In our user-testing process, prototypes went through iterations that incorporated feedback from users regarding acceptability, usability, and functionality. We sought to address the needs of survivors and physicians while balancing the capacity and infrastructure available for a lifelong intervention via our health system partners.

**Conclusions:**

In partnership with childhood cancer survivors, family physicians, and health system partners, we elucidated the barriers and enablers to accessing guideline-recommended surveillance tests and designed a multifaceted solution that will support survivors and their family physicians. The next step is to evaluate the intervention in a pragmatic randomized controlled trial.

## Introduction

Approximately 80% of childhood cancer survivors will develop a serious, life threatening, or disabling late effect from their curative treatment by age 45 [1]. Cardiomyopathy and subsequent malignant neoplasms (particularly breast or colon cancer) are among the late effects with the greatest impact on both serious morbidity and premature mortality. North American guidelines [2,3] include recommendations for cancer surveillance (eg, mammography or breast magnetic resonance imaging [MRI] in women with a history of chest radiation and colonoscopy in survivors treated with abdominal or pelvic radiation) and echocardiographic assessment in survivors at risk for cardiac dysfunction due to exposure of the heart to radiation or anthracycline chemotherapy.

Unfortunately, most adult survivors of childhood cancer do not receive the recommended surveillance, placing them at risk for preventable harm [4-6]. Our recent study of over 10,000 North American adult survivors of childhood cancer revealed that only 13%, 37%, and 41% of high-risk individuals were currently adherent to recommended breast, colorectal, and cardiac screening, respectively.

A systematic review assessed the effectiveness of interventions promoting adherence to surveillance guidelines in adult survivors of childhood cancer [7]. Only 3 trials assessed interventions to improve uptake of our targeted tests (colonoscopy, breast imaging, and echocardiograms) in survivors of childhood cancer [8-10]. These interventions were found to be resource intensive and thus do not represent sustainable programs at a population level. Furthermore, interventions in these trials relied on survivors sharing educational materials with their primary care provider rather than purposefully educating primary care professionals on the needs of childhood cancer survivors [8,9]. This review, coupled with a recent review of interventions in routine risk populations to improve uptake of cancer screening [11], indicated that personalizing the invitation for surveillance [12], ensuring primary care endorsement [13], and providing reminders [14] could each play an incremental role in increasing completion of recommended tests. In summary, a 2-pronged intervention that engages both survivors and their primary care clinician has the potential to significantly reduce morbidity and mortality by improving adherence to surveillance guidelines.

Therefore, working with cancer health system partners, we embarked on a rigorous design process for an intervention to address cancer surveillance for late effects among childhood cancer survivors. Our approach used behavioral science and design thinking. Behavioral science allowed us to employ relevant theories of behavior change to understand the factors that might influence surveillance adherence. We also used methods from design thinking, a “human-centered approach to innovation—anchored in understanding customer's needs, rapid prototyping, and generating creative ideas” [15]. Design thinking has recently been applied in health care to address patient experiences, clinical outcomes, and health care spending [16-18]. We used these methods to gain a deeper, more empathic understanding of the experience of adult survivors of childhood cancer. This paper outlines how we used these 2 methodologies in a multistep process in the design of a provincial surveillance and support system for childhood cancer survivors.

## Methods

### Overview

**Figure 1:**
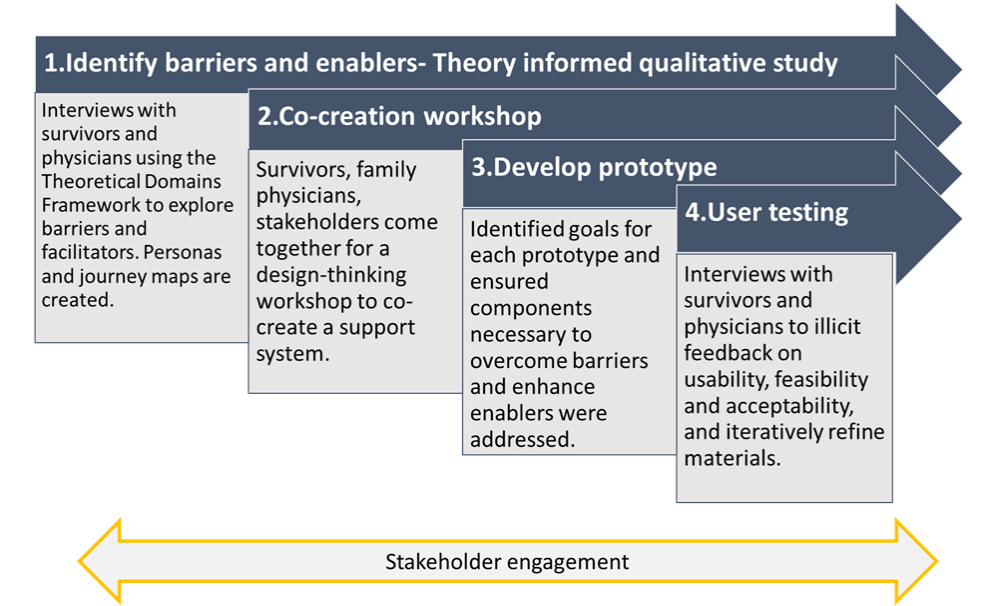
Four-step approach to design a childhood cancer surveillance and support system.

We used a 4-step approach to design a childhood cancer surveillance and support system to facilitate completion of surveillance tests (echocardiograms, breast MRI, mammograms, and colonoscopy) among childhood cancer survivors (Table 1 and Figure 1). We chose a multidisciplinary method to incorporate different perspectives and approaches that were relevant to the design of an intervention. Our complementary approach can improve the fit between evidence-based theories (ie, behavioral theory like the Theoretical Domains Framework [19]), the strategies used to implement them (ie, implementation science [20]), and their implementation contexts (ie, using design thinking [15]). Furthermore, design thinking goes further than traditional barrier and facilitator assessment by embedding users more deeply in the process, thereby enhancing the usability and usefulness of the intervention.

**Table 1:** Stages of development of childhood cancer surveillance system.

Intervention stage and description		Objective	Methods used	Products produced
Discover: theory-informed qualitative study [21]		Identification of key barriers, facilitators, needs, and challenges the intervention must address	Qualitative interviews Thematic analysis Behavioral theory—Theoretical Domains Framework Design thinking	Personas and journey maps Theoretical Domains Framework Behavior change techniques to be addressed in intervention
**Design and build^a^**				
	One-day workshop	Creation of guiding principles to help summarize and easily refer to features of the intervention identified as central to achieving its objectives	Design thinking	Personas and journey maps Intervention components “worksheet” Validation of concept
	Development of prototype	N/A^b^	N/A	Design of prototypes: survivor invitation letter; survivor information kit; website; survivor reminder letters; physician information letter
	User testing design^a^	All intervention components evaluated in detail and optimized from survivor and physician perspective	Two rounds of iterative user testing Think aloud methodology	Iterative changes to protypes developed with each round Documented changes
Evaluate: pragmatic randomized controlled trial^c^		Intervention evaluated in real-life context(s), modified to improve implementation in future contexts	N/A	N/A

^a^We met regularly with stakeholders to review the emergent intervention design.

^b^N/A: Not applicable.

^c^To be completed in 2022-2023 (not reported in this paper).

In Step 1, we conducted a qualitative study of childhood cancer survivors and family physicians to explore intervention components that are essential regarding accessing evidence-based, high-yield surveillance tests. Semistructured interviews with adult survivors of childhood cancer who were eligible for 1 or more of the surveillance tests of interest (but had not attended a specialized survivor clinic in over 5 years) were completed. Survivors were asked to specify the details of their primary care provider or family physician, who were then invited to participate in an interview. We have reported on a previous analysis using the Theoretical Domains Framework (TDF) [22,23] and behavior change techniques [24,25] to identify influences on accessing surveillance tests among survivors [21]. The TDF proposes a comprehensive, theory-informed approach frequently used by implementation scientists to identify the determinants of behavior and behavior change in health care professionals and patients [23]. The TDF ensures that the full set of potentially important determinants of behavior, including those that may be especially relevant to the completion of surveillance, such as emotion, social norms, and beliefs about capabilities, are considered. Furthermore, it offers a strategy for mapping key determinants of behavior to relevant behavior change techniques to include in an intervention. In this way, the TDF can provide key insights into selecting the necessary intervention components and tailoring those components for different survivors [26].

In this study, we used thematic analysis [27] supported by the TDF to identify specific barriers and enablers to accessing and providing surveillance tests as they relate to intervention components. Transcripts were independently coded line-by-line by 2 research team members (authors JS and NS). Codes were then thematically analyzed and described in terms of how they could guide and shape the intervention. Next, initial themes were examined and refined to confirm that the themes characterized the data set as a whole and no themes were missed. Data collection and analysis continued iteratively until saturation was achieved—that is, until no new ideas were introduced during subsequent interviews [28]. To expand our knowledge of how our results can inform our intervention, we drew upon behavior change techniques associated with the TDF domains [24,25] that the intervention should address.

Then, using design thinking methodology, we created personas [29] and journey maps based on our interviews and analysis. Personas are fictional characters that represent an archetype character. They helped identify the user's needs and wishes and enabled the team to engage and empathize during the design process. Journey maps are a visualization of the process that a person goes through to accomplish a goal. We used this tool to dissect the process a survivor goes through from discharge from pediatric care to various life stages. It helped the team to think about the different moving parts of follow-up for a childhood cancer survivor and assisted with illuminating areas of potential interest. The personas guided the design and content decisions and addressed specific behavior techniques to be tackled in the intervention. For example, when addressing the behavior change technique “reduce negative emotions,” we ensured our ideas and content were consistent with the personas.

**Figure 2:**
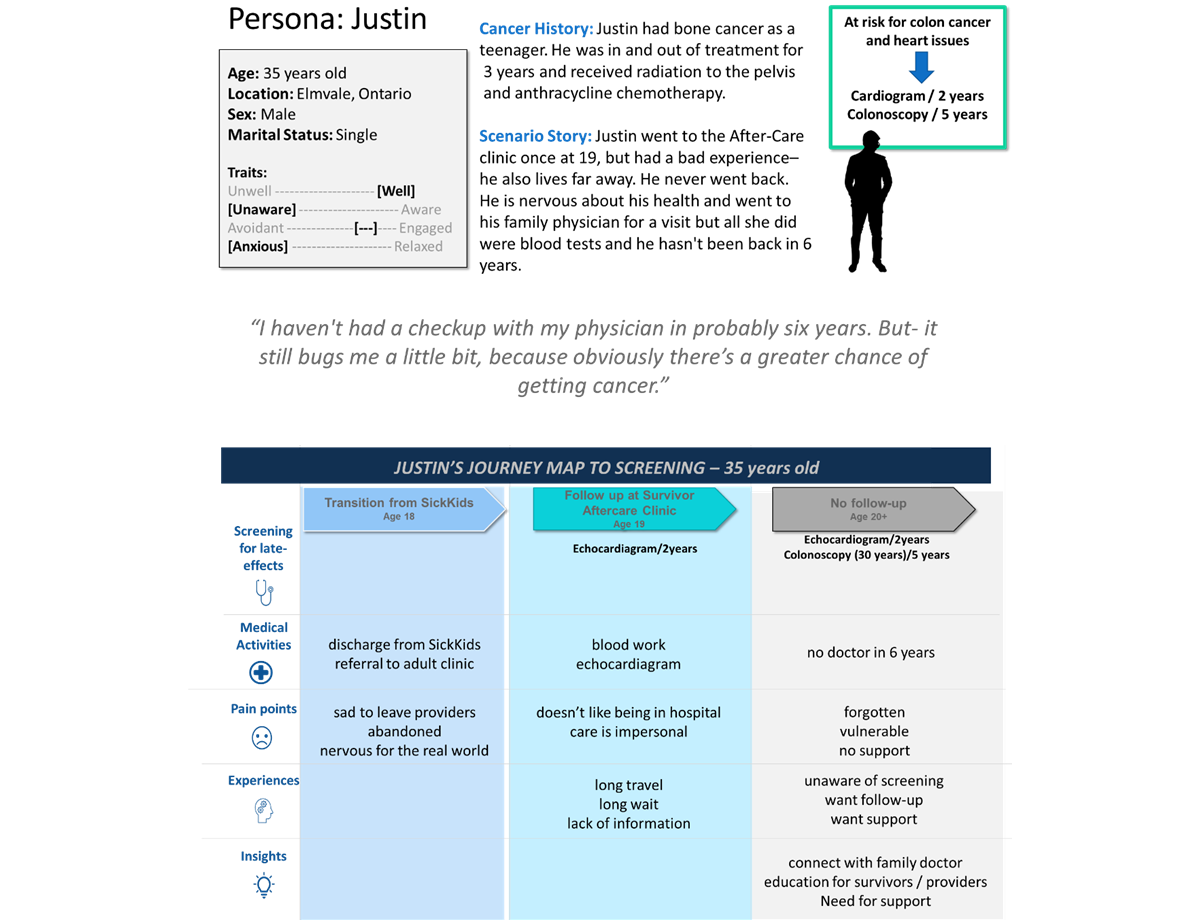
Example of personas and journey maps.

In Step 2, we organized a 1-day cocreation workshop in July 2020 that brought together childhood cancer survivors, primary care physicians, and cancer health system partners. The objective of the workshop was to validate findings from Step 1 and elicit ideas on the design and content of the surveillance system. Our team worked with Pivot Design Group, a design firm, to develop and facilitate the workshop. Select childhood cancer survivors and physicians who participated in Step 1 were invited to participate in this workshop. We purposively invited a diverse group of survivors who varied in age, location, and screening recommendations. After presenting the findings from Step 1, including personas and journey maps, we divided participants into 3 groups. Each group was assigned a persona and tasked with developing solutions regarding accessing surveillance for their persona (Figure 2). We wanted to evoke, understand, and overcome pain points through idea generation and develop a long-term solution together. At the end of the session, each group presented their solutions and then engaged in a discussion with the larger group. The session and breakout groups were recorded, and data was extracted into the following broad categories: (1) content, (2) functionality, (3) design, and (4) barriers. Data was then compared across the different breakout groups to identify similarities and differences.

In Step 3, upon consultation with survivors, physicians, and health system partners, our team developed the following prototypes: (1) survivor invitation letter, (2) survivor information kit, (3) website, (4) survivor reminder letters, and (5) physician information letter. Based on Steps 1 and 2, the following actions were taken to ensure the content was effective. First, we identified goals for each prototype and made sure that all components necessary to overcome barriers and enhance enablers (behavior change techniques [24,25] from Step 1) were addressed. We also incorporated principles of design, including decisions on font, colors, and logos. Finally, we made all decisions while considering the emotional impact that engaging with these materials would have on childhood cancer survivors.

In Step 4, we used the user-centered design methodology to iteratively refine the intervention materials and gain feedback on the usability, feasibility, and acceptability of the materials. Childhood cancer survivors who were eligible for the surveillance tests of interest (echocardiogram, mammogram/breast MRI, or colonoscopy) but had not attended a specialized survivor clinic in over 5 years were invited from 3 such clinics in Ontario (some survivors were reinvited for an interview from Step 1). Family physicians were recruited through social media and family medicine networks. In the first round, prototypes were low fidelity, and screen share technology was used to show the materials to participants. Materials were then updated based on feedback, and the design was improved with the help of a designer. In the second round of testing, materials were mailed to survivors and opened during the interview. We used the “think aloud” method wherein participants were encouraged to share thoughts, likes, and dislikes as they went through the materials. Interviews were recorded and data extracted, synthesized, and thematically analyzed to understand the user experience. The team then reviewed results and made design decisions iteratively.

### Ethics Approval

This multistep and multisite study involved several components of research ethics approval. Step 1 of our study was approved by Clinical Trials Ontario (project ID 1906), with The Hospital for Sick Children's Research Ethics Board acting as the Board of Record. Steps 2 and 4 were conducted following the Women's College Hospital's Quality Improvement approval process. Institutional approvals were sought at all relevant participating sites where applicable. All survivors and physicians gave informed consent to participate in the study.

## Results

### Step 1: Qualitative Study

We interviewed 30 survivors and 7 family physicians (Table 2). Childhood cancer survivors were keen to learn more about their risk for late effects and surveillance recommendations. Concurrently, it was deemed crucial that survivors' emotions, including cancer-related anxiety, would be addressed in the intervention. We learned that information on late effects and accessing surveillance could help empower survivors with the knowledge and tools necessary to complete tests but that the intervention must also reduce the burden of remembering when tests are due and scheduling appointments. Based on the barriers and enables identified, as well as the experience and knowledge of our team, we developed 4 survivor personas, 1 family physician persona, and corresponding journey maps (see Figure 2 for an example). Personas differed regarding their family context (single versus married with children), location (small town versus city) and emotional state (anxious versus not). The survivors' personas included a grading on a scale of traits such as feeling unwell versus well, unaware of late effects versus aware, avoidant of health care versus engaged, and anxious versus relaxed.

Our analysis of interviews with family physicians revealed that barriers to supporting childhood cancer survivors in their practice included physicians' unfamiliarity with long-term follow-up care guidelines for childhood cancer survivors, time constraints in each patient interaction, and limited support in unpacking the unique needs of childhood cancer survivors. Physicians vocalized that personalized information about their patient's needs would ensure that they could support their patient in accessing surveillance tests. Based on these interviews, we developed a surveillance concept that involved 3 components: (1) help reconnect childhood cancer survivor to the health system; (2) provide information on recommended screening tests; and (3) remind survivors of upcoming screening tests (Figure 2).

**Table 2:** Characteristics of childhood cancer survivors (N=30) and family physicians (N=7).

Respondents and their characteristics			Values
**Childhood cancer survivors**			
	Age (years), mean (SD)		41 (10.5)
	**Frequency of doctor visits, n (%)**		
		≤Once a year	27 (90)
		>Once a year	1 (3)
		Undetermined/very infrequently	2 (7)
	**Location, n (%)**		
		Urban	22 (73)
		Rural	8 (27)
	**Gender, n (%)**		
		Female	18 (60)
		Male	12 (40)
	**Highest level of education attained, n (%)**		
		Less than high school	1 (3)
		High school	1 (3)
		College/university/graduate	28 (94)
	**Type of cancer, n (%)**		
		Lymphoma	10 (33)
		Leukemia	7 (24)
		Neuroblastoma	2 (7)
		Wilms tumor	3 (10)
		Bone tumor	5 (16)
		Liver tumor	3 (10)
**Family physicians**			
	Age (years), mean (SD)		45 (14)
	Years in practice, mean (SD)		17 (4)

### Step 2: Workshop

**Figure 3:**
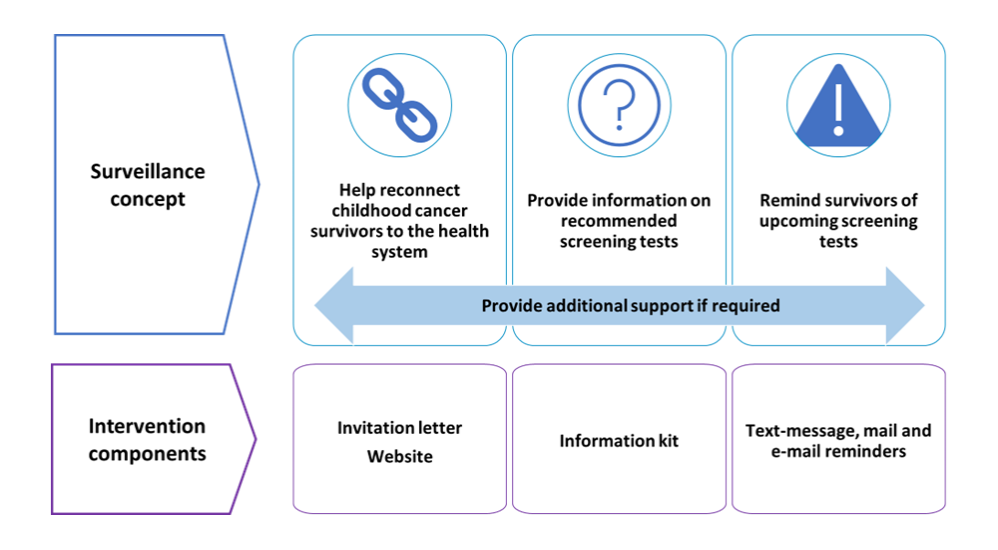
Childhood cancer survivor surveillance concept and corresponding intervention components.

A total of 6 childhood cancer survivors, 3 family physicians, and 3 health system partner stakeholders attended the workshop. Stakeholders represented relevant provincial health system partner organizations. The workshop validated our surveillance concept (Figure 3) and provided key insights regarding the development of the intervention components. In terms of the invitation letter, we learned from survivors that receiving a letter regarding their cancer could be stressful and anxiety provoking. Interestingly, survivors were not concerned about privacy in receiving a letter identifying them as a childhood cancer survivor in the mail. The importance of involving the family physician in the intervention was highlighted. Family physicians expressed a desire to receive concise, clear information regarding the individual patient's history and the next steps required. Survivors preferred different options regarding methods of communication (ie, mail, email, text message). Survivors communicated the importance of additional information, if required, and a website for more information. Finally, survivors appreciated the idea of an “opt-in” program where the initial outreach only had general information and then they could choose if they wanted to receive personalized information regarding their risks and screening recommendations, including periodic reminders.

### Step 3: Prototype Development

Protypes were developed by establishing goals for each prototype, ensuring the content addressed the specific barriers and enablers from the qualitative study, the behavior change techniques identified [21] were implemented as intervention components (Table 3), and the intervention components from the workshop were incorporated. For example, in the introductory letter and information kit for the survivors, we addressed the following: (1) personalized information on how to complete surveillance tests and information about health consequences of late effects, delivered in a impactful manner; (2) prompts/cues to perform the required tests and supports to enable surveillance while conserving mental resources; (3) persuasive information on the health benefits of surveillance; and (4) supports to reduce fear of cancer and negative emotions linked to surveillance.

In recognition that survivors may feel anxiety or fear when receiving a letter about their childhood cancer, it was important to choose a color scheme that would help survivors feel at calm and safe. We chose a blue as a dominant color because it has been shown to be associated with trust and confidence [30,31]. To ensure buy-in from survivors, we knew it would be important to include recognizable logos. Therefore, to lend credibility to the program, we included logos to link it to an already established and recognized organization.

**Table 3:** Prototype development.

Item	Goals	Discovery phase			Design choices
		Domains of the Theoretical Domains Framework [19,22]	Behavior change techniques mapped from the Theoretical Domains Framework that were addressed^a^ [24]		
Survivor invitation letter	Generate awareness of late effects and surveillance guidelines Reconnect childhood cancer survivors to the health system Confirm identity Offer personalized information Determine preferred method of contact (email, mail, text message) Confirm primary care provider	Knowledge (of late effects) Emotion (fear of cancer)	Biofeedback Instruction on how to perform behavior Information about antecedents Information about health consequences Information about social and environmental consequences Reduce negative emotions	Include information on how to obtain surveillance Include information about health consequences of late effects Advise on ways to reduce fear of cancer and negative emotions linked to surveillance	
Survivor information kit	Provide tailored information To enlist action: share with primary care provider	Beliefs about consequences (of surveillance) Intention (to complete surveillance tests for late effects)	Information about health consequences Salience of consequences Information about social and environmental consequences Anticipated regret Information about emotional consequences Goal setting (behavior) Information about health consequences Self-incentive	Provide information on health benefits of surveillance in an effective and memorable manner and awareness of possible regret if surveillance is not performed Provide information about emotional benefit of completing surveillance and tips regarding self-incentive if surveillance is performed	
Website	Increase legitimacy of program A place to find more information if desired	See knowledge, emotion, beliefs about consequences	N/A^b^	N/A	
Survivor reminder letters	Ensure survivors do not forget about surveillance test To enlist action: reach out to physician to book test	Memory, attention, and decision-making (reminders)	Prompts/cues Conserving mental resources	Include prompts/cues to perform surveillance Enable surveillance completion while conserving mental resources	
Physician information letter	Education on patient history and surveillance recommendations To enlist action: contact survivor and use their electronic medical record to schedule reminders	Knowledge (of late effects)	Instruction on how to perform behavior Information about antecedents Information about health consequences Information about social and environmental consequences	Include information on patient’s cancer history, risk of late effects, and surveillance recommendations	

^a^An international consensus project identified a list of 93 behavior change techniques as elemental components of interventions. It was developed to help intervention designers, researchers, and theorists in the development and evaluation of theory-based interventions. Published linkage of behavior change techniques to Theoretical Domains Framework domains is based on triangulating relationships found in published studies and by expert consensus (see [32]).

^b^N/A: not applicable.

### Step 4: User Testing

#### Round 1

In the first round of user testing, we interviewed 5 survivors and 2 physicians (Table 4). Key insights and areas for improvement are highlighted regarding each prototype.

##### Invitation Letter

Survivors did not find the letter overwhelming and were glad to learn about late effects. They appreciated our acknowledgment that learning this information could be stressful and that we provided a point of contact for additional support. There was some confusion related to the program flow (ie, how to access surveillance tests), for which adjustments were made. Survivors expressed hesitancy in joining the surveillance program, with our analysis suggesting that this could stem from the survivor lacking a family physician, not feeling at risk for late effects, feeling uncertain of the benefits of the program, or experiencing fear related to surveillance tests. In the next iteration, we addressed these issues by highlighting benefits (physical and emotional benefit) and clear information on accessing a family doctor.

**Table 4:** Characteristics of the participants in user testing (N=11).

Characteristics		Round 1 (n=5)	Round 2 (n=6)
**Gender, n (%)**			
	Male	3 (60)	2 (33)
	Female	2 (40)	4 (66)
Age (years), mean (SD)		35 (8)	37 (10)
**Location, n (%)**			
	Urban	3 (60)	3 (50)
	Rural	2 (40)	3 (50)

##### Website

Survivors were glad to have a website with more information on late effects and the program. However, some were uncertain about how to sign up. Survivors also found the website stale and boring; thus, in the next iteration, we strove to make the website aesthetically pleasing while maintaining a sense of importance and urgency.

##### Information Kit

Survivors' interest in the program increased after they saw the information kit. This demonstrated that it would be beneficial for survivors to have a better understanding of the information kit when then received the introductory letter. There was a range of desire for information; therefore, we decided that the website would be a good forum to include additional information. There was also some confusion on what to do next and the role of the survivor in the program. Changes were made to highlight the program flow, and we highlighted the importance of bringing this information kit to their family doctor. Some survivors felt overwhelmed by the responsibility of taking on these surveillance tests. In the next iteration, we included empowering language and emphasized that their family physician would help them through the process.

##### Reminders

Survivors wished to receive reminders via email. They also wanted more information on when their test was due instead of a simple message that they were due for a test. They felt that these reminders looked official and would encourage them to book a test. Some expressed that they wished that these tests could be booked without a family doctor, but that was not something we could address in the confines of this provincial health system in Ontario.

##### Physician Information Letter

We interviewed 2 physicians in the first round, and the information letter was well received by both. These physicians were glad to know that the survivors would also be receiving their screening recommendations. They found the letter clear and concise but requested additional information on accessing tests and what to write on the requisition. They requested reminder letters to ensure that they could help their patients keep on track with their surveillance tests, and this was developed for the next round of testing. It was important for them to see the logos of provincial health organizations on the letter.

#### Round 2

In the second round of interviews, we spoke with 6 childhood cancer survivors and 6 family physicians (Table 4). Overall, the design of the materials was well received and there were minimal suggestions for changes.

##### Invitation Letter

Survivors appreciated the opportunity to choose to learn more information on late effects and screening recommendations. They felt empowered with the new knowledge and supported to complete the recommended surveillance. They were enthusiastic to learn more through the program website and felt that this gave the program legitimacy. Some survivors questioned the credibility of the program and wondered how their personal health information was collected. Changes were made to explain the organizations that were supporting this program and why they had access to their personal information.

##### Information Kit

Survivors found the kit clear and concise and felt it would be useful to bring in to show their family physician.

##### Reminders

Reminders were appreciated and thought of as an essential component of the program.

##### Physician Information and Reminder Letter

Some physicians found the tone of the letter to be prescriptive rather than collaborative. They also requested more information on their patient’s cancer diagnosis and treatment history so they could have a more informed conversation with their patient. Physicians highlighted that the possibility that survivors did not receive information on late effects and surveillance should be made clear. It was important for physicians that the patient was also engaged in the program and received a letter. They wanted the patients to be partners in their care and share responsibility of initiating contact with the physician to order the surveillance tests.

Finally, the feasibility of this study was carefully considered in the context of the infrastructure of our stakeholders in this phase. For example, some users wanted a highly tailored information kit that included many follow-up recommendations, but this was not feasible with existing data sets that were leveraged for this intervention. Survivors also requested an online portal where they could view their screening recommendations; however, stakeholders were concerned with privacy and were not interested in creating and maintaining a website with this type of information.

## Discussion

Our design process used both behavioral theory and design thinking to develop a complex intervention aimed at increasing adherence to surveillance guidelines for late effects among childhood cancer survivors. We began with a discovery stage, where we identified important barriers, such as the burden of managing care and lack of knowledge among both survivors and physicians through a behavioral theory–led process. Survivors' emotions, including cancer and surveillance–related anxiety, required careful consideration. It was further emphasized by survivors that the support system must help with reducing the burden of remembering when tests are due and scheduling appointments. Personas and journey maps enabled our team to empathize and design with different types of survivors in mind (eg, different stages of life and geographical location). During our design and build phase, we tailored the prototypes to best empower survivors, give them sufficient and clear information, address their fears, and provide them with necessary support, directions, and reminders to promote access to surveillance tests.

The result of this process is a design of a centralized system to identify high-risk survivors and provide them and their family physicians with personalized information about recommended surveillance and periodic reminders. Our surveillance and support system builds on existing recommendations put forth by the National Cancer Policy Board of the Institute of Medicine and the National Research Council to design systems of care that are responsive to the long-term needs of childhood cancer survivors as they transition from pediatric to adult care, improve awareness of late effects and their implications among survivors, and augment professional education and training regarding late effects for primary care clinicians [33]. Our next step is to implement and evaluate this intervention in a pragmatic randomized controlled trial with an embedded process evaluation.

Our discovery and iterative design process described in this paper led to new insights for this population and may help the development of other surveillance systems aimed to increase screening. For example, our discovery process highlighted the importance of engaging both physicians and patients simultaneously in the intervention so they can both be partners in care, a component missing from most previous interventions for childhood cancer survivors [7]. We also demonstrated that by addressing and supporting emotional needs and empowering individuals to take control of their health, outreach to a population with a complex health history is not only feasible but also welcomed. This barrier has not been thoroughly described or addressed in previous interventions aimed at improving adherence to surveillance guidelines in survivors of childhood cancer [7].

This study adds to the growing literature on the design of interventions using user-centered methodology and behavioral [34-38] and psychological [39,40] theory. As per our experience, we found these 2 methodologies to be compatible and beneficial. We were able to design for childhood cancer survivors in a user-centered empathetic fashion while also infusing rigor and systematic thinking by incorporating behavioral theory. There is growing need to address complex system problems in a manner that is compassionate and user-focused while also relying on insights from behavioral science at the various stages of design and evaluation.

To maximize the likelihood of implementation, we made sure to engage relevant stakeholders who will ultimately be rolling out the intervention if it is shown to be effective when tested in a pragmatic controlled trial. Therefore, during the design and build phase, we had to ensure that the design of our intervention would be compatible with operationalization. For instance, survivors voiced preference on which organization they would prefer to receive surveillance information from; however, we had constraints based on privacy and could not align our design with this preference. We also had to rely on the existing outreach infrastructure; therefore, we could not accommodate physicians’ preference to receive information on late effects and surveillance recommendations via fax. Other elements that were expressed as important during the cocreation workshop, such as a nurse navigator, were removed from the design because they were costly and incompatible with creating a sustainable intervention. This process has highlighted the importance of designing an intervention in tandem with ongoing conversations with necessary stakeholders who will be delivering the program.

This work highlights the feasibility and value of using behavioral science and design thinking in the development of a scalable and long-term health system approach to address the surveillance needs of childhood cancer survivors. However, there are some limitations to our work. First, using a design thinking approach enabled us to develop a deeper understanding of survivors’ and primary care clinicians’ needs and communicate them in an actionable way, but it also had constraints. For example, we had to balance survivors’ and physicians’ wishes with the capacity and infrastructure of our health system partners. Additionally, this methodology was not straightforward; at times, it was challenging to prioritize divergent feedback from user groups, and multiple rounds of feedback and iterations can be time consuming. Additionally, since feedback was gathered during user testing, we had to be mindful of carefully considering implications before adding new features. Finally, we were only able to work with survivors who responded to our invitations, and the extent that these insights are generalizable to the general childhood cancer survivor community is unknown.

In this article, we present an example of intervention design using behavioral theory and design thinking. In partnership with survivors, family physicians, and health system partners, we have elucidated actionable barriers and enablers related to completion of guideline-recommended surveillance by adult survivors of childhood cancer and designed a multifaceted solution that will support survivors and their family physicians. Further directions include evaluating this intervention through a pragmatic randomized controlled trial.

## Abbreviations

CIHR: Canadian Institutes of Health Research
MRI: magnetic resonance imaging
POGO: Pediatric Oncology Group of Ontario
